# Assessment of *BPV-1* Mediated Matrix Metalloproteinase Genes Deregulation in the In Vivo and In Vitro Models Designed to Explore Molecular Nature of Equine Sarcoids

**DOI:** 10.3390/cells11081268

**Published:** 2022-04-08

**Authors:** Przemysław Podstawski, Katarzyna Ropka-Molik, Ewelina Semik-Gurgul, Marcin Samiec, Maria Skrzyszowska, Zenon Podstawski, Tomasz Szmatoła, Maciej Witkowski, Klaudia Pawlina-Tyszko

**Affiliations:** 1 Department of Animal Molecular Biology, National Research Institute of Animal Production, Krakowska 1 Street, 32-083 Balice, Poland; ewelina.semik@iz.edu.pl (E.S.-G.); tomasz.szmatola@iz.edu.pl (T.S.); klaudia.pawlina@iz.edu.pl (K.P.-T.); 2 Department of Animal Reproduction, Anatomy and Genomics, University of Agriculture in Kraków, Mickiewicza 24/28, 30-059 Krakow, Poland; zenon.podstawski@urk.edu.pl; 3 Department of Reproductive Biotechnology and Cryoconservation, National Research Institute of Animal Production, Krakowska 1 Street, 32-083 Balice, Poland; marcin.samiec@iz.edu.pl (M.S.); maria.skrzyszowska@iz.edu.pl (M.S.); 4 Center for Experimental and Innovative Medicine, University of Agriculture in Krakow, Rędzina 1c Street, 30-248 Krakow, Poland; 5 Institute of Veterinary Medicine, University Centre of Veterinary Medicine JU-AU, Mickiewicza 24/28, 30-248 Krakow, Poland; mawitkow@gmail.com; 6 Szpital Koni Służewiec, Puławska 266, 02-684 Warszawa, Poland

**Keywords:** matrix metalloproteinase, sarcoid, skin neoplasia, NGS, cDNA microarray

## Abstract

Matrix metalloproteinases (*MMP*s) represent a family of enzymes capable of biocatalytically breaking down the structural and functional proteins responsible for extracellular matrix (ECM) integrity. This capability is widely used in physiological processes; however, imbalanced MMP activity can trigger the onset and progression of various pathological changes, including the neoplasmic transformation of different cell types. We sought to uncover molecular mechanisms underlying alterations in transcriptional profiles of genes coding for *MMP*s, which were comprehensively identified in equine adult dermal tissue bioptates, sarcoid-derived explants, and ex vivo expanded adult cutaneous fibroblast cell (ACFC) lines subjected to inducible oncogenic transformation into sarcoid-like cells. The results strongly support the hypothesis that the transcriptional activity of *MMP* genes correlates with molecular modifications arising in equine dermal cells during their conversion into sarcoid cells. The alterations in *MMP* transcription signatures occurs in both sarcoid tissues and experimentally transformed equine ACFC lines expressing *BPV1-E4^E1* transgene, which were characterized by gene up- and down-regulation patterns.

## 1. Introduction

Matrix metalloproteinases (MMPs) are proteins responsible for the disassembly of the extracellular matrix (ECM), which allows for cell migration and the release of various signaling factors. MMPs can regulate growth factors, whose activity is required to mediate different processes at a given tissue development stage [1,2]. Depending on their specific substrate, these proteins can be divided and assigned into six classes: collagenases, gelatinases, stromelysins, matrilisins, membrane-type MMPs, and other MMPs [1]. A wide variety of MMPs are involved in many physiological processes that are indispensable for remodeling of the tissue environment, e.g., wound healing. Importantly, the destabilization of their expression pattern in the tissue can lead to the promotion of many pathological changes such as intestinal inflammation in humans or recurrent airway obstruction and laminitis in horses, and various tumors (i.e., lung cancer, liver metastases, and gastric carcinomas) [3]. Moreover, *MMP*s are believed to incur the initiation and progression of neoplastic transformation processes by breaking down the ECM proteins. Related to the degradation function of ECM is the ability of cancer cells to metastasize. The breakdown of ECM and the various growth factors, cytokines, and chemokines released as a result of this process make metalloproteinases involved in every step of metastasis, starting with the induction of epithelial to mesenchymal transition (EMT), resistance to anoikis, supporting the angiogenesis process, and activating cancer cells invasion and colonization of new tissues. Therefore, metalloproteinases are considered to be one of the major supportive factors for cancer. The large influence of metalloproteinases on metastasis, a process that is the most common cause of death from cancer, makes them an important aspect of research in the context of cancer development [4,5]. However, molecular pathways regulating the transcriptional activity of particular genes in this family have not been precisely recognized so far. Some *MMP*s have been found to be over-expressed in neoplastic tissues, and others have been shown to be over-expressed in healthy tissues as a result of a natural response to inflammatory processes [3].

**Figure 1:**
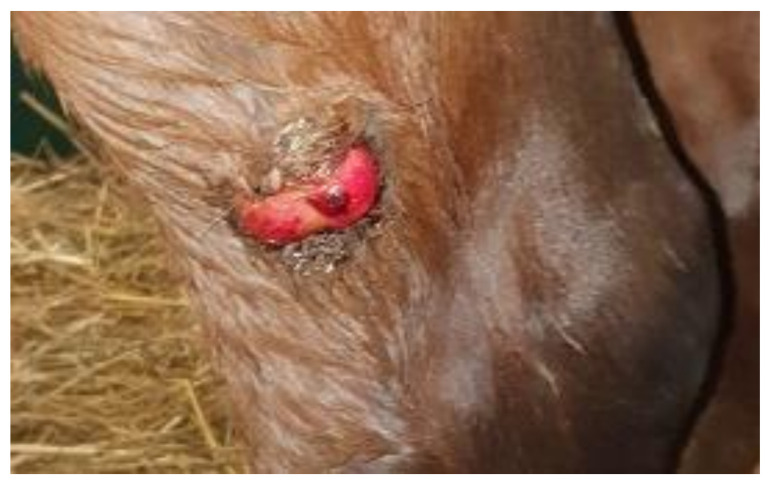
Example of sarcoid lesion—sarcoid surgically removed from a two-year-old horse (noble half-blood horse); sarcoid was localized on gaskin (by Witkowski M).

The most common skin tumor in the *Equidae* family is the sarcoid (Figure 1). It manifests itself in many forms, which vary according to the extent of severity, and it can decrease the quality of animal life. It has been established that sarcoids do not metastasize [6]. There is general agreement that bovine delta-papillomaviruses induce sarcoids, i.e., bovine papillomaviruses types 1 and 2, and possibly 13 (*BPV1*, *BPV2*, and *BPV13*) [7,8], but the molecular nature of the BPV oncoprotein-associated tumor formation process is not entirely understood.

Therefore, research targeted to comprehensively unravel the role of MMP expression and deregulation in the oncogenic transformation of equine dermal cells into sarcoids and their spread is imperative. Consequently, the objective of the current study was to explore the transcriptional profile of a panel of *MMP* genes in healthy equine skin and sarcoid tumors as well as in expanded adult cutaneous fibroblast cells (ACFCs) transfected with *BPV1-E4^E1* fusion gene, which can be considered as an in vitro model for one of the development steps of sarcoid disease.

## 2. Materials and Methods

### 2.1. MMP Genes Selection

**Figure 2:**
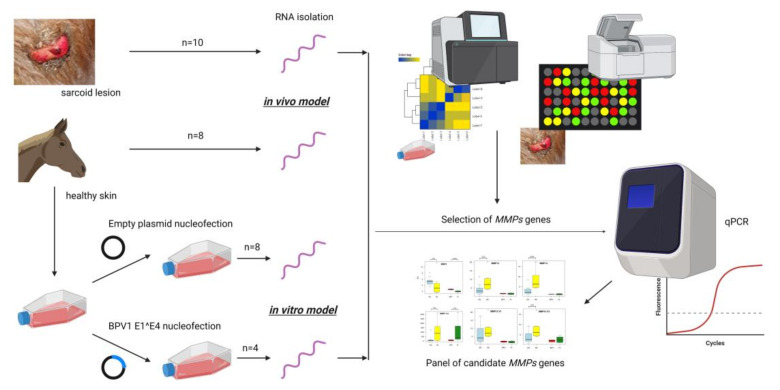
The experimental schedule. Created with BioRender.com (accessed on 31 March 2022) [11].

The experimental workflow is presented in Figure 2. The high-throughput analysis of transcriptomic signatures [9] was used to identify the *MMP* genes for further analyses based on applying cDNA microarrays to compare the gene expression patterns in healthy skin and sarcoid tissue samples. Additionally, using the RNA-seq technique, a panel of studies was accomplished to estimate the *MMP* gene expression profiles in both *BPV-E4^E1* transfected ACFC lines and their counterparts transfected with empty vectors as a negative control group [10]. Considering both data sets generated (designated as GSE83430 and GSE193906), genes that belonged to the MMP family and their corresponding fold change values were achieved. The data obtained from microarrays were reanalyzed in a way similar to previous work [9], however without final filtering of genes by fold-change (*p*-value < 0.05). The RNA-seq data were obtained via sequencing of cDNA libraries on NextSeq 500 Illumina platform (Illumina) using NextSeq 500/550 High Output KIT v 2.5 (75 cycles) and according to protocol. The libraries were prepared using TruSeq RNA Kit v2 kit (Illumina) and evaluated using Qubit fluorimeter (Invitrogen, Thermo Fisher, Santa Clara, CA, USA). The obtained reads were mapped to EquCab3 reference genome (STAR software v 2.7.8). Next, the mapped reads were annotated and counted to specific gene thresholds using Ensembl gtf file version 100 (via htseq-count software v 0.13.5). Differential expression analysis was performed with the use of Deseq2 software (significance showed as *p*-value < 0.05 after multiple testing corrections).

### 2.2. Sample Preparation

The biological samples comprised (i) equine sarcoid explants (*n* = 10) collected during standard veterinarian tumor removal, (ii) healthy skin bioptates (*n* = 8) collected at a local slaughterhouse, and (iii) ex vivo expanded ACFC lines. For all samples, the presence of *BPV*1 and 2 DNA was validated by PCR (AmpliTaq Gold™ 360 Master Mix; Thermo Fisher; according to the protocol) using specific primers as previously reported by Teifke et al. [12]. Skin bioptates scoring positive for *BPV*1 and 2 DNA were excluded from further experiments. The established ACFC lines were allotted to two groups: control cell lines transfected with an empty vector (pcDNA4/TO/myc-his/B; *n* = 8) and cell lines transfected with *BPV1-E4^E1* gene constructs (*pcDNA4/E4^E1/TO/myc-his/B*; *n* = 4). The detailed procedures applied to establish ACFC lines and transfectant cells described by Podstawski et al. [10]. In short, fibroblast lines derived postmortem from equine skin were nucleofected with two plasmids included in the T-REx kit (Invitrogen, Waltham, MA, USA, Thermo Scientific): *pcDNA^TM^ 6/TR* and *pcDNA™ 4/TO/myc-His/B* (with or without (negative control) ligated *BPV1-E1^E6* gene construct). Nucleofection procedure was performed with Amaxa^TM^ Normal Human Dermal Fibroblast– Adult (NHDF-Adult) Nucleofector^TM^ Kit (Lonza, CELLLAB, Warsaw, Poland). Positively transfected cell lines were obtained during antibiotic selection with 200 µg/mL zeocin (Invitrogen) and 6 µg/mL blasticidin S (Thermo Scientific, Waltham, MA, USA), which was held for one week.

### 2.3. Quantitative PCR Analysis

**Figure 3:**
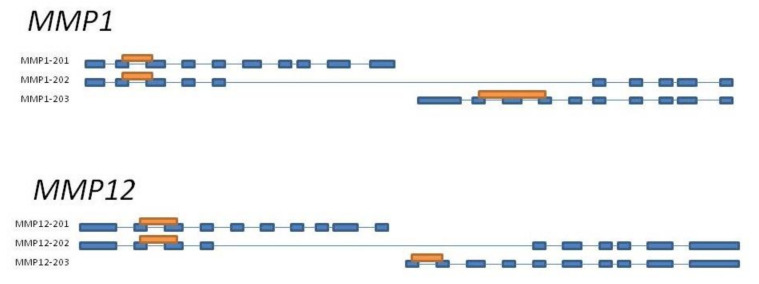
Schematic presentation of primers designed for *MMP1* and *MMP12* genes to amplify all splicing variants. *MMP–*201–203 are splicing variants according to Ensembl accession number (*MMP1*-ENSECAG00000023733; *MMP12*-ENSECAG00000019445); primer amplification region is presented as an orange rectangle, and blue boxes represent exons.

The conditions and parameters of qPCR method were thoroughly specified in our previous report [13]. Briefly, the RNA was isolated with PureLink RNA Mini Kit (Invitrogen, Thermo Fisher, according to the protocol). The quality and quantity of obtained RNA were checked using TapeStaion 2200 (Agilent Technologies, Santa Clara, CA, USA) and RNA Screen Tape (Agilent Technologies, Santa Clara, CA, USA). cDNA strand synthesis was performed with High-Capacity cDNA reverse transcription kit (Applied Biosystems, Thermo Scientific, according to the protocol). The qPCR was carried out with AmpliQ 5× HOT EvaGreen qPCR Mix Plus (ROX) (BIOTUM, Novazym, Poznań, Poland, according to the protocol) and specific primers (Table 1, Figure 3). Each sample was performed in three replications. Obtained results were calculated with the ΔΔCT method [14] based on two endogenous controls: β-actin (*ACTB)* and ubiquitin B (*UBB)* [15].

### 2.4. Statistical Analysis

The obtained RQ values were firstly tested for distribution purposes with a Shapiro–Francia normality test followed by Mann–Whitney U-test to assess the inter-group variability. Both tests were carried out using R software v 4.1 [16].

## 3. Results and Discussion

**Figure 4:**
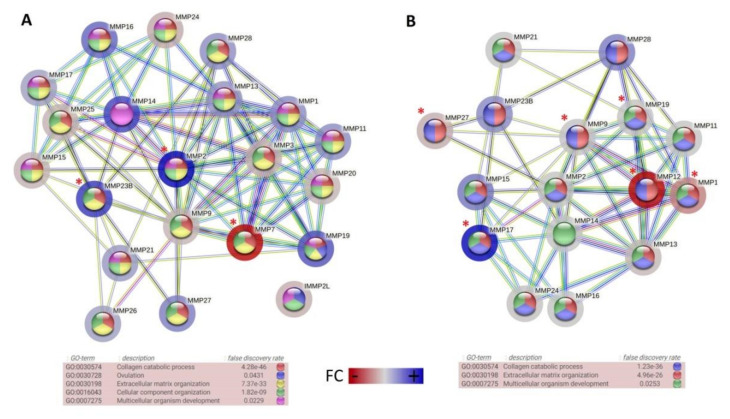
The Gene ontology terms and interaction between sets of *MMP* genes identified using cDNA microarray in sarcoid and healthy skin tissues (**A**) and using RNA-seq approach in control ACFC lines nucleofected with empty vector or with *BPV1-E4^E1* transgene (**B**); significant differentially expressed genes were marked with asterisks (*p*-value < 0.05); the fold change (FC) directions were marked with colors (String software [17] *Equus Caballus* reference).

The present study was designed to determine changes in the *MMP* gene expression profiles that can be positively correlated with the development of equine sarcoids. For this purpose, to comprehensively evaluate the molecular patterns of sarcoid-dependent neoplasmic transformation, the in vivo and in vitro models have been applied. To comparatively examine the transcriptomic signatures of *MMP* genes, the expression profile in equine sarcoid tissue samples and *BPV1-E4^E1* nucleofected ACFC-derived tumor cells were compared to healthy dermal tissues and ACFC lines not subjected to oncogenic transformation. First, the high throughput data was used to detect the *MMP* genes expressed in the investigated groups of cells and tissues and to demonstrate significant differences between them. The comparative analysis of *MMP* genes between sarcoid and healthy skin tissue samples allowed us to detect a total of 21 genes belonging to the *MMP* family, out of which three representatives (*MMP2*, *MMP7*, and *MMP23B*) underwent significantly differential expression patterns (Figure 4A). In turn, the whole transcriptome sequencing-mediated analysis of *BPV1-E4^E1* transgenic equine ACFC-derived neoplastic cell lines and their counterparts nucleofected with empty vectors revealed the expression of 16 *MMP* family genes, out of which six members (*MMP1*, –*9*, –*12*, –*17*, –*19*, and –*27*) showed significantly differential expression (Figure 4B).

Next, the expression profiling using qPCR confirmed modifying the transcriptional activities for *MMP* genes representing both in vitro and in vivo models of sarcoid-dependent oncogenic transformation. Additionally, for two genes (*MMP1* and *MMP12*), the splice variant analysis was also performed to prove the possible involvement of transcripts encoding different *MMP* isoforms in the processes of either transgenically induced neoplastic transformation in *BPV-E4^E1* nucleofected ACFCs into sarcoid-like cells or pathophysiological conversion of dermal tissues into sarcoids.

**Figure 5:**
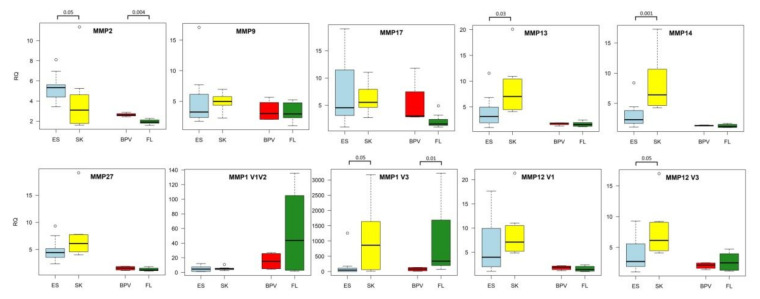
The differences in *MMP* gene expression levels between analyzed groups of equine sarcoids (ES), skin (SK) samples, control ACFC lines nucleofected with empty vector (FL), and ACFC lines nucleofected with *BPV-E4^E1* transgene (BPV) (R software v 4.1) [16].

Expression of several *MMP* genes was already investigated in equine sarcoids in terms of ECM remodeling [18]. It was hypothesized that ECM remodeling is a pivotal step of etiopathogenesis of sarcoid-dependent neoplasia of equine dermal tissues that provides clear evidence for the occurrence of aberrant expression of *MMP* genes. Martano et al. have also proven that the altered transcriptional activities of *MMP* and *TIMP* genes can lead to imbalance between collagen synthesis and degradation processes and, consequently, can result in skin neoplasia progression [18]. In turn, the study results by Yuan et al. [16] have confirmed that *MMP1*, *–2*, and *–9* genes were up-regulated in sarcoid tumors and their over-expression was highly associated with the invasion fibroblast-derived sarcoid cells. Compared to the published data, we provide strong evidence of significant differences in the expression levels of *MMP2* and *MMP9* genes, which turned out to be up-regulated in both the ex vivo and in vitro model of sarcoid-specific neoplasmic transformation (Figure 5). Furthermore, an augmented transcriptional activity of *MMP17* gene was also observed in dermal sarcoid samples and *BPV1-E4^E1* nucleofected ACFC derivatives. *MMP17* belongs to genes, whose enhanced expression has been found to be related to onset and progression of inflammatory processes. Therefore, it’s up-regulation may be a biomarker of inflammation evoked in sarcoid tissues and oncogenically transformed cells [19].

The whole transcriptome analysis on the in vitro model reflecting *BPV1* infection in equine dermal fibroblast cells confirms the remarkable deregulation of *MMP* genes in *BPV1-E4* and *BPV1-E4^E1* transgenic ACFC neoplastic cell lines [10]. In 2008, Yuan et al. [20] evaluated the impact of *BPV1* on the transcriptional profile of equine fibroblast cell lines. They pinpointed that the *MMP1* gene was one of the most deregulated gene associated with the virus infection. Taking into account different splice isoforms of the *MMP1* gene, the present research has identified a significant down-regulation of this gene in sarcoid tissue samples and nucleofected ACFC lines. Such modification was the most evident for V3 variant designated as ENSECAT00000025715.2 (fold changes equal to −5.90 and −12.26; *p*-values < 0.05 and < 0.01, respectively) (Figure 5). Moreover, as depicted in Figure 5, a similar expression profile was detected for all analyzed *MMP12* isoforms, which were characterized by significantly and insignificantly diminished levels of V3 variant termed as ENSECAT00000020742.2 in sarcoid tissue explants (fold change equal to −1.95; *p*-value < 0.05) and *BPV1-E4^E1* nucleofected ACFC lines (FC 1.31; *p*-value ≥ 0.05), respectively. This can indicate that *BPV1* infection contributes to diminished *MMP1* and *MMP12* expression levels. Moreover, different involvement of individual splice variants may suggest the importance of their individual roles in the neoplastic process.

Interestingly, transcription of three other genes—*MMP13* (*p*-value < 0.05), *MMP14* (*p*-value < 0.001), and *MMP27* (*p*-value ≥ 0.05)—was down-regulated in sarcoid tissue samples as compared to healthy skin bioptates. Observed *MMP27* down-regulation contrasts with the results of other studies performed on tumors, where *MMP27* mRNA was equally expressed in normal breast and breast cancer tissues [21]. These findings can support the hypothesis that MMP27 protein can play an important role in oncogenic transformation in a variety of tumor types. In turn, down-regulation of *MMP13* and *MMP14* genes can be one of the most important reasons for sarcoids’ very or negligibly low capability to metastasize, since *MMP14* gene silencing brings about the negative control of cell migration [22]. In contrast, the *MMP13* gene is considered to be a poor prognostic biomarker related to cell migration in several cancer types (i.e., colorectal cancer and breast cancer) [23,24].

## 4. Conclusions

To conclude, the present report strongly supports the hypothesis that the transcriptional activity of *MMP* genes is correlated with molecular modifications arising in equine dermal cells during their molecular conversion into sarcoid cells. The alterations in the *MMP* transcriptomic signatures that were shown to occur in sarcoid tissues and transformed equine ACFC lines expressing *BPV1-E4^E1* transgene were characterized by both *MMP*s up- and down-regulation patterns. Moreover, for *MMP1* and *MMP12* genes, significant involvement of selected splice variants may suggest that only selected isoforms are crucial for the growth and development of sarcoids.

## Figures and Tables

**Table 1:** Specific primer sets used for amplification of MMPs cDNA. We investigated specific splice isoforms and exons spans.

Gene	Accesion Number	Primers	Product Length [bp]	Localisation	Splice Variants	Splice Variants Accesion Number
*MMP1* V1V2	ENSECAG00000023733	F: GCTGAAAGTGACTGGGAAGC R: GGTCCACATCTGCTCTTGGT	179	Exon 2 and 3	2/3	ENSECAT00000022856.2 ENSECAT00000067257.1
*MMP1* V3	ENSECAG00000023733	F: CCCAAGTGGGAACGAAATAA R: ATTCCATTGGGTCCATCAAA	224	Exon 12 and 14	1/3	ENSECAT00000025715.2
*MMP2*	ENSECAG00000000953	F: TCCCTTTCCTCTTCAACGGC R: CCGTATTTGCCGTCCTTGTC	112	Exon 4	2/2	ENSECAT00000044147.1 ENSECAT00000004278.3
*MMP9*	ENSECAG00000013081	F: CGTGTTTCCCTTCACCTTCG R: GGTCGTAGTTGGCGGTAGT	141	Exon 6	3/3	ENSECAT00000056131.1 ENSECAT00000014089.3 ENSECAT00000068636.1
*MMP12* V1V2	ENSECAG00000019445	F: TGGACATGATGCACAGACCT R: CAGCCTCGCCTGTGTTAATC	222	Exon 2 and 3	2/3	ENSECAT00000002326.3 ENSECAT00000077256.1
*MMP12* V3	ENSECAG00000019445	F:GATCTGCAAGGGACGAGGAT R:CACCTCCACAGTGTCAGAGT	194	Exon 11 and 12	1/3	ENSECAT00000020742.2
*MMP13*	ENSECAG00000005506	F:GCTCCGAGAAATGCAGTCTT R: GCCATTTGAGAGTTCGAGGG	140	Exon 2	1/1	ENSECAT00000007039.2
*MMP14*	ENSECAG00000008351	F: CATGATCTTCTTCGCTGAGGG R: GGTGTCGCCTCCAATGTTG	109	Exon 4	1/1	ENSECAT00000008899.3
*MMP17*	ENSECAG00000013201	F: CACTACGCCCTCAAAGTCTG R: AGGGATAGCGGTCATTGTGG	118	Exon 4	2/2	ENSECAT00000013924.2 ENSECAT00000013881.2
*MMP27*	ENSECAG00000019404	F: CGGTGACTGGAAAACTGGAT R: CCCTTGGAAATCTTGGTGAA	238	Exon 2 and 3	1/1	ENSECAT00000020635.3

## Data Availability

Not applicable.
